# Telocytes reduce oxidative stress by downregulating DUOX2 expression in inflamed lungs of mice: Telocytes reduce oxidative stress in inflamed lungs of mice

**DOI:** 10.3724/abbs.2022017

**Published:** 2022-02-16

**Authors:** Haihong Tang, Tao Liang, Yile Zhou, Huihui Ju, Dongli Song, Hao Fang

**Affiliations:** 1 Department of Anesthesiology Zhongshan Hospital Fudan University Shanghai 200032 China; 2 Department of Anesthesiology Jinshan Hospital Fudan University Shanghai 201508 China; 3 Department of Anesthesiology Shanghai East Hospital Tongji University Shanghai 200120 China; 4 Zhongshan Hospital Institute for Clinical Science Shanghai Xuhui Central Hospital Zhongshan-Xuhui Hospital Zhongshan Hospital of Fudan University Shanghai 200032 China; 5 Department of Anesthesiology Minhang Hospital Fudan University Shanghai 201199 China

**Keywords:** telocyte, miR-146a-5p, DUOX2, oxidative stress, cAMP-response element-binding protein 1

## Abstract

Telocytes (TCs), a novel type of interstitial cells, have been found to participate in tissue protection and repair. In this study, we investigated the antioxidative effects of TCs in inflamed lungs of mice. Acute respiratory distress syndrome (ARDS) mice were used as models of inflamed lungs of mice. Gene sequencing was used to screen the differentially expressed miRNAs in TCs after lipopolysaccharide (LPS) stimulation. AntagomiR-146a-5p-pretreated TCs were first injected into mice, and antioxidant activity of TCs was estimated. TCs, RAW264.7 cells, and MLE-12 cells were collected for the detection of expressions of NOX1–4, DUOX1–2, SOD1–3, GPX1–2, CAT, Nrf2, miR-146a-5p, and miR-21a-3p after LPS stimulation. Silencing miRNAs were delivered to examine the involved signaling pathways. Oxidative stress was examined by measuring malondialdehyde (MDA) levels. We found that microRNA-146a-5p and microRNA-21a-3p were upregulated in TCs after LPS stimulation. ARDS mice that were preinfused with TCs had lower lung tissue injury scores, lung wet-dry ratios, white blood cell counts in alveolar lavage fluid and lower MDA concentrations in lung tissue. However, in antagomiR-146a-5p-pretreated ARDS mice, the infusion of TCs caused no corresponding changes. After LPS stimulation, DUOX2 and MDA concentrations were downregulated in TCs, while DUOX2 was restored by antagomiR-146a-5p in TCs. Dual-luciferase reporter assay confirmed that CREB1 was downregulated by miR-146a-5p, while DUOX2 was downregulated by CREB1, which was confirmed by treating TCs with a specific CREB1 inhibitor. This study demonstrates that LPS stimulation upregulates miR-146a-5p in TCs, which downregulates the CREB1/DUOX2 pathway, resulting in a decrease in oxidative stress in cultured TCs. TCs reduce LPS-induced oxidative stress by decreasing DUOX2 in inflamed lungs of mice.

## Introduction

Acute respiratory distress syndrome (ARDS) is an acute clinical symptom characterized by diffuse alveolar cell damage and severe injury of the respiratory alveolo-capillary barrier followed by non-cardiogenic pulmonary edema and hypoxic dyspnea, due to widespread inflammation in the lungs [ 1– 4]. Enhanced oxidative stress is an important mechanism underlying the development of ARDS [ 5, 6]. It was reported that ARDS patients have higher levels of hydrogen peroxide in their breath than healthy subjects [ 7, 8]. In pulmonary tissue, oxidative stress reduces the formation of alveolar surfactants, leading to a collapse of alveoli. Antioxidant treatments reduce the leakage of bronchoalveolar lavage and mitigate pulmonary edema in LPS-induced lung injury [9].

In the structure of lung telocytes (TCs), a novel type of interstitial cells were described soon after their discovery in 2010 [ 10, 11]. These peculiar cells, characterized by uncommon cellular extensions called telopodes, were characterized in many organs [ 12, 13]. Among these localizations, the presence of TCs has been reported in the myocardium [14], gastrointestinal tract [ 15, 16], lungs [ 17, 18] and kidneys where TCs were shown to exert protective effects in acute kidney injury [19], infected lung [20] as well as infarcted myocardium [21]. Lung TCs have been extensively investigated by genomic and proteomic studies, which reveal that TCs can be differentiated from other cell types found in the lung parenchyma, such as fibroblasts, endothelial cells, pneumocytes, airway cells, mesenchymal stem cells (MSCs), and lymphocytes [ 22– 24]. The therapeutic roles of TCs in the respiratory tract were hypothesized from the beginning, and one important role in the reduction of inflammation in experimental astma was demonstrated after transplantation of TCs, which could improve allergen-induced asthma by obviously inhibiting airway inflammation and airway hyper-responsiveness preclinically, with the down-regulation of Th2-related cytokine IL-4, transcription factor GATA-3 and Th2 cell differentiation, and the up-regulation of Th1-related cytokine IFN-γ, transcription factor T-bet and Th1 cell proliferation in asthma [25]. Our previous study demonstrated that TCs increase MSC migration in the attempt to treat experimental acute lung injury [26]. Moreover, co-cultivation of TCs with other cells, like stem cells, exert important effects. We found that VEGF, IL-6 and some chemokines stimulated by IL-6 signalling are secreted by cardiac TCs and overexpressed in co-cultures with cardiac stem cells (CSCs). The expression levels of MIP-2 and MIP-1α were increased by 2 folds and 4 folds, respectively, when TCs were co-cultured with CSCs, while the expression of IL-2 did not significantly differ between TCs and CSCs in mono culture but significantly decreased by 2 folds in the co-culture system. These data suggest that the TC secretome plays a modulatory role in stem cell proliferation and differentiation [27], and this may also be important for the lung.

In our previous study, we found that exogenous TCs protect against LPS-induced pulmonary injury by reducing cytokine levels [20] and promoting angiogenesis [28]. In the *in vitro* experiments, TCs were co-cultured with primary human dermal microvascular endothelial cells (HDMECs) as well as mouse fibroblast cells (L929), and the results showed that TCs significantly reduced LPS-induced oxidative stress [29], indicating that they have antioxidative features. However, the antioxidant role of TCs in LPS-induced lung injury has not been extensively explored. A previous study in 2019 on a lung telocyte cell-line transformed with Simian vacuolating virus 40 small and large T antigen (SV40) showed that the proliferative capacity of TCs ^SV40^ declined with the increase of LPS or TNFα concentration, similar to the responses of primary TCs [30]. Therefore, we hypothesized that lung TCs ^SV40^ may be a useful tool in the development of challenging new therapies.

MicroRNAs (miRNAs), which are small noncoding ribonucleic acid molecules, regulate target genes by inhibiting mRNA translation or promoting mRNA degradation. Moreover, miRNAs have been found in the extracellular vesicles released by TCs, both *in vitro* and *in vivo* [ 31, 32]. It has been reported that miRNAs regulate reactive oxygen species (ROS) in cardiovascular disease [33]. MicroRNA-146a-5p regulates DUOX2 expression through both signal transducer and activator of transcription 1 (STAT1) [34] and cAMP-response element-binding protein 1 (CREB1) [35]. Previous study in our lab demonstrated that LPS stimulation increases the expressions of miR-146a-5p and miR-21a-3p in TCs [28] which takes part in the regulation of target genes by inhibiting mRNA translation or promoting mRNA degradation [ 36, 37].

In the present study, we aimed to investigate the antioxidative effects of TCs in the inflamed lungs of mice, with a special focus on the underlying mechanisms of noncoding RNAs functioning on the enzymes involved in oxidative stress in TCs.

## Materials and Methods

### Reagents and antibodies

LPS was purchased from Sigma (St Louis, USA). Antibodies against CREB1 (ab31387) and phosphorylated CREB1 (ab32096) were purchased from Abcam (Cambridge, USA). Antibody against DUOX2 (NB110-61576ss) was purchased from NOVUS (Centennial, USA). Specific inhibitors of CREB1, KG-501 (HY-103299) and 666-15 (HY-101120) were purchased from MedChem Express (MCE, Monmouth Junction, USA), Lipofectamine RNAiMAX was purchased from Thermo Fisher Scientific (Rockford, USA). Bicinchoninic acid assay (BCA) kit, RIPA lysis buffer, and loading buffer were purchased from Beyotime (Shanghai, China).

### Cell cultures

Mouse lung TCs were kindly provided by Dr Xiangdong Wang (Biomedical Research Center, Zhongshan Hospital, Fudan University, Shanghai, China), and cells were transfected with SV40 large and small T antigen to constructed TCs ^SV40^ [38]. TCs ^SV40^ cells were cultured in Dulbecco’s modified Eagle’s medium/F12 (DMEM/F12; Thermo Fisher Scientific) supplemented with 10% fetal calf serum (FBS; Thermo Fisher Scientific). Mouse RAW264.7 and MLE-12 cells were obtained from Zhongqiao Tech (Shanghai, China). All the cells were cultured in DMEM/F12. HEK293T cells (CRL-11268; ATCC, Manassas, USA) were cultured in DMEM high glucose medium (Hyclon, Gaithersburg, USA) for the Dual luciferase reporter assay [39].

### Cell transfection

Cell transfection was performed using Lipofectamine RNAiMAX (Thermo Fisher Scientific) according to the manufactory’s protocol. In brief, 50 nM antagomiR-146a-5p (Cat. 20000158; Ribo Biotech, Guanzhou, China) was prepared in a serum-free and antibiotic-free medium. After transfection, TCs were supplied with full growth medium until confluence. For animal experiments, TCs (1×10 ^6^ cells) were freshly prepared in 100 μL PBS.

### Animal experiments

Twenty-four male C57BL/6 mice (8 weeks old, 22–25 g) were obtained from the Shanghai SLAC Animal Company (Shanghai, China) and kept in a pathogen-free condition. The mice were housed in standard air-conditioned rooms (22–24°C) under a 12/12 h light/dark cycle, with free access to food and water. Mice were randomly divided into four groups: the control group, LPS stimulation group, TCs treatment group challenged with LPS, and miR-146a-5p-silenced TCs treatment group challenged with LPS. The mouse ARDS model was established by perfusing mice with LPS (5 mg/kg) via the trachea. TCs (1×10 ^6^ cells) transfected with antagomiR-146a-5p or not, were administered via the tail vein 2 h before the LPS stimulation. The animals were sacrificed, and the lungs and bronchoalveolar lavage fluid were collected 24 h after the establishment of the mouse ARDS model.

All animal experments were performed following the bioethics guidelines and were approved by the Institutional Animal Care and Use Committee of Zhongshan Hospital, Fudan University (Shanghai, China).

### Assessment of lung injury

To collect white blood cells in bronchoalveolar lavage fluid, mice lungs were perfused with ice-cold phosphate-buffered saline (PBS). After centrifugation at 3000 *g*, white blood cells were counted under a microscope (JapanBX43; Olympus, Tokyo, Japan). The left lung was fixed in 10% formalin solution and embedded in paraffin for hematoxylin and eosin (H&E) staining. The right lung was collected to assess edema by the ratio of wet weight/dry weight (W/D) of the lung after incubation in a 60°C oven for 48 h. The lung injury was evaluated using a modified lung injury histological scoring system [38]. In brief, lung injury was scored on a scale of 0–4 using the average score of the following items: (I) alveolar capillary congestion, (II) hemorrhage, (III) infiltration of neutrophils into the airspace or the vessel wall and thickness of the alveolar wall, and (IV) alveolar wall thickness/hyaline membrane formation. A score of 0 represents normal findings, and scores between 1–4 represent the following levels of lung injuries: 1, mild lung injury (<25% lung involvement); 2, moderate lung injury (25%–50% lung involvement); 3, severe lung injury (50%–75% lung involvement); and 4, very severe lung injury (>75% lung involvement). The cytokines levels of IL-1, IL-6, and TNF-α in bronchoalveolar lavage fluid were detected by Bio-Plex Pro Mouse Cytokine 23-Plex Assay on the Bio-Rad MAGPIX Multiplex Reader (Bio-Rad, Hercules, USA).

### Reverse transcription-polymerase chain reaction

Total RNA was extracted from cultured TCs using Trizol reagent (TaKaRa, Shiga, Japan). MicroRNAs were reversely transcripted with a Bulge-Loop miRNA qRT-PCR Starter kit (Ribo Biotech), and mRNAs were reversely transcripted to complementary DNA (cDNA) using a PrimeScript RT Reagent kit with gDNA Eraser (TaKaRa). Both miRNAs and mRNAs were amplified on an ABI 7500 sequence detection system (Applied Biosystems, Foster, USA). The expression levels of miR-21a-3p (Cat. MQPS0002615) and miR-146a-5p (Cat. MQPS0002462) were normalized with *U6* and mRNAs expressions were normalized with *GAPDH*. Primers used in the present study were synthesized by Sangon Biotech (Shanghai, China) as listed in Table 1.

**Table:** **Table1** Sequences of primers used in this study

Gene	Forward (5′→3′)	Reverse (5′→3′)
*NOX1*	GGTTGGGGCTGAACATTTTTTC	TCGACACACAGGAATCAGGAT
*NOX2*	TGTGGTTGGGGCTGAATGTC	CTGAGAAAGGAGAGCAGATTTCG
*NOX3*	CAACGCACAGGCTCAAATGG	CACTCTCGTTCAGAATCCAGC
*NOX4*	CCAAATGTTGGGCGATTGTGT	CAGGACTGTCCGGCACATAG
*DUOX1*	AAAACACCAGGAACGGATTGT	AGAAGACATTGGGCTGTAGGG
*DUOX2*	AAGTTCAAGCAGTACAAGCGAT	TAGGCACGGTCTGCAAACAG
*SOD1*	GGAACCATCCACTTCGAGCA	CCCATGCTGGCCTTCAGTTA
*SOD2*	TTCTGGACAAACCTGAGCCC	GTCACGCTTGATAGCCTCCA
*SOD3*	CCTTCTTGTTCTACGGCTTGC	TCGCCTATCTTCTCAACCAGG
*GPX1*	AGTCCACCGTGTATGCCTTCT	GAGACGCGACATTCTCAATGA
*GPX2*	AAGTGTGACGTCAATGGGCA	TGAGGGAGAACGGGTCATCA
*CAT*	AGCGACCAGATGAAGCAGTG	TCCGCTCTCTGTCAAAGTGTG
*GAPDH*	GTTCAACGGCACAGTCAAG	GCCAGTAGACTCCACGACAT

### Western blot analysis

Cultured cells were lysed in RIPA buffer containing protease inhibitors (Roche, Indianapolis, USA), phosphatase inhibitors (Beyotime), and phenylmethanesulfonyl fluoride (PMFS; Beyotime). Total protein concentration was determined using the bicinchoninic acid assay kit (Beyotime). Samples (30 μg) were subject to SDS-PAGE and then transferred onto polyvinylidene fluoridewere (PVDF) membranes. Mebranes were blocked with 5% bovine serum albumin in Tris-buffered saline containing 0.1% Tween 20. The membranes were then incubated with primary antibody at 4°C for overnight. On the second day, the membranes were further incubated with an HRP-conjugated anti-rabbit IgG secondary antibody (1:2000; CST-7074; Cell Signaling Technology, Danvers, USA) for 1 h. Finally, target protein signals were visualized with Omni-ECL Femto Light Chemiluminescence kit (EpiZyme, Shanghai, China) on a Fluorescent Image Detection System (Tanon-52Multi; Tanon, Shanghai, China). The related signals were quantified using ImageJ software and normalized with GAPDH. The information of all the antibodies are shown as follows: antibodies against JAK1 (CST-3344), JAK2 (CST-3230), JAK3 (CST-8827), TYK2 (CST-14193), STAT1 (CST-14994), STAT2 (CST-72604), STAT3 (CST-30835), STAT4 (CST-2653), STAT5 (CST-94205), STAT6 (CST-5397), phosphorylated JAK1 (CST-74129), phosphorylated JAK2 (CST-8082), phosphorylated JAK3 (CST-5031), phosphorylated TYK2 (CST-68790), phosphorylated STAT1 (CST-7649), phosphorylated STAT2 (CST-4441), phosphorylated STAT3(Try705) (CST-9145), phosphorylated STAT3 (Ser727) (CST-9134), phosphorylated STAT5 (CST-4322), phosphorylated STAT6 (CST-9316), and GAPDH (CST-5558) were purchased from Cell Signaling Technology.

### Dual luciferase reporter assay

The PHY-881 reporter vector (Hanyin Biotechnology, Shanghai, China) was used to construct the luciferase reporter vector plasmids PHY-881-WT-CREB1-3′-UTR and PHY-881-Mut-CREB1-3′-UTR. The plasmids CREB1-WT or CREB1-Mut were co-transfected with miR-146a-5p mimic (Cat. 10000158; Ribo Biotech) or negative control (Cat. 0000001; Ribo Biotech) into human embryonic kidney (HEK293T) cells using Lipofectamine 2000 reagent (Thermo Fisher Scientific). A dual-luciferase reporter assay system (Promega, Madison, USA) was used to assess luciferase activity after 24 h of transfection. Relative firefly luciferase activity was normalized to renilla luciferase activity.

### Malondialdehyde (MDA) assay

Malondialdehyde levels in TCs and culture medium were measured using malondialdehyde (MDA) assay kit (S0131; Beyotime) according to the manufacturer’s instructions. Briefly, culture medium or cell lysates were incubated in MDA working solution at boiling temperature for 15 min. The MDA concentration was determined by measuring the absorbances at 532 nm, and normalized with protein concentrations in culture medium or cell lysates, respectively.

### Statistical analysis

Data were presented as the mean±standard deviation. The data were analyzed using the GraphPad Prism software (GraphPad Software, LaJolla, USA). One-way analysis of variance (ANOVA) was used for comparison. *P*<0.05 was considered statistically significant.

## Results

### AntagomiR-146a-5p deminishes the lung protection role of exogenous TCs in LPS-induced ARDS

**Figure 1:**
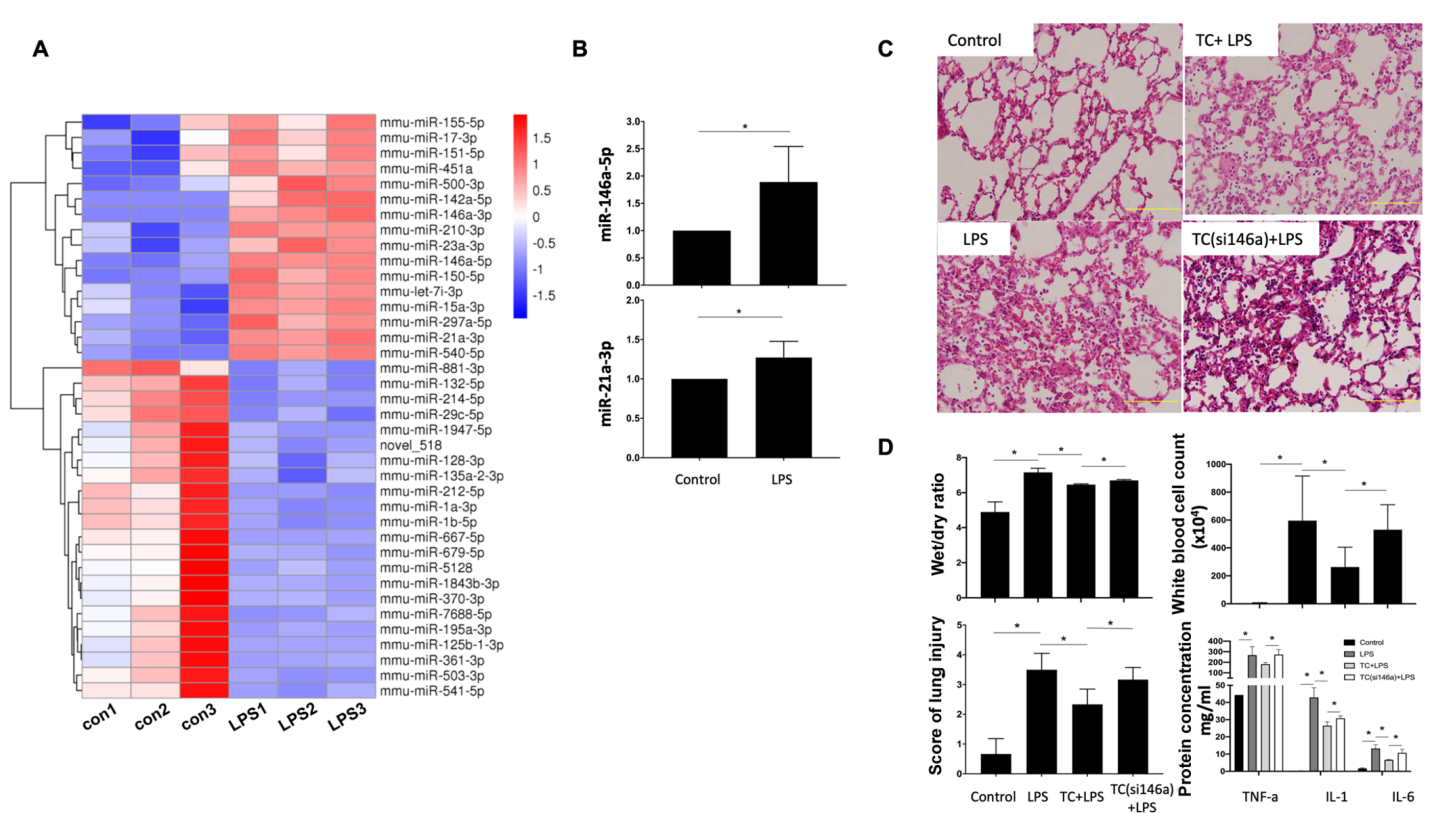
Figure1 MiR-146a-5p in TCs plays an essential lung protection role in the mouse ARDS model (A) Heat map of differentially-expressed miRNAs in cultured TCs stimulated with LPS. (B) LPS significantly increased the expressions of miR-146a-5p and miR-21a-3p in TCs. (C,D) Compared with ARDS mice, TC-treated ARDS mice had a reduced lung tissue injury score, a lower W/D ratio of the lung, fewer white blood cells, and lower concentrations of cytokines in alveolar lavage fluid. However, when being silenced with antagomiR-146a-5p, TCs failed to exert any protective effects on LPS-stimulated ARDS mice. Data are shown as the mean±standard deviation from six independent experiments (n=6). *P< 0.05.

In our previous studies, microarray sequencing was performed to identify differential exppressions of genens [28]. Here we showed that LPS significantly increased the expressions of miR-146a-5p and miR-21a-3p in TCs ( Figure 1A,B). The ARDS model was established by LPS instillation [ 36, 37]. TCs reduced the score of lung tissue injury ( Figure 1C,D), W/D ratio of the lung, number of white blood cells, and cytokine concentration in alveolar lavage fluid in ARDS mice ( Figure 1D). However, when being transfected with antagomiR-146a-5p, TCs failed to exert any protective effects on LPS-stimulated ARDS mice ( Figure 1C,D).

### AntagomiR-146a-5p decreases oxidative stress both *in vitro* and *in* *vivo*

**Figure 2:**
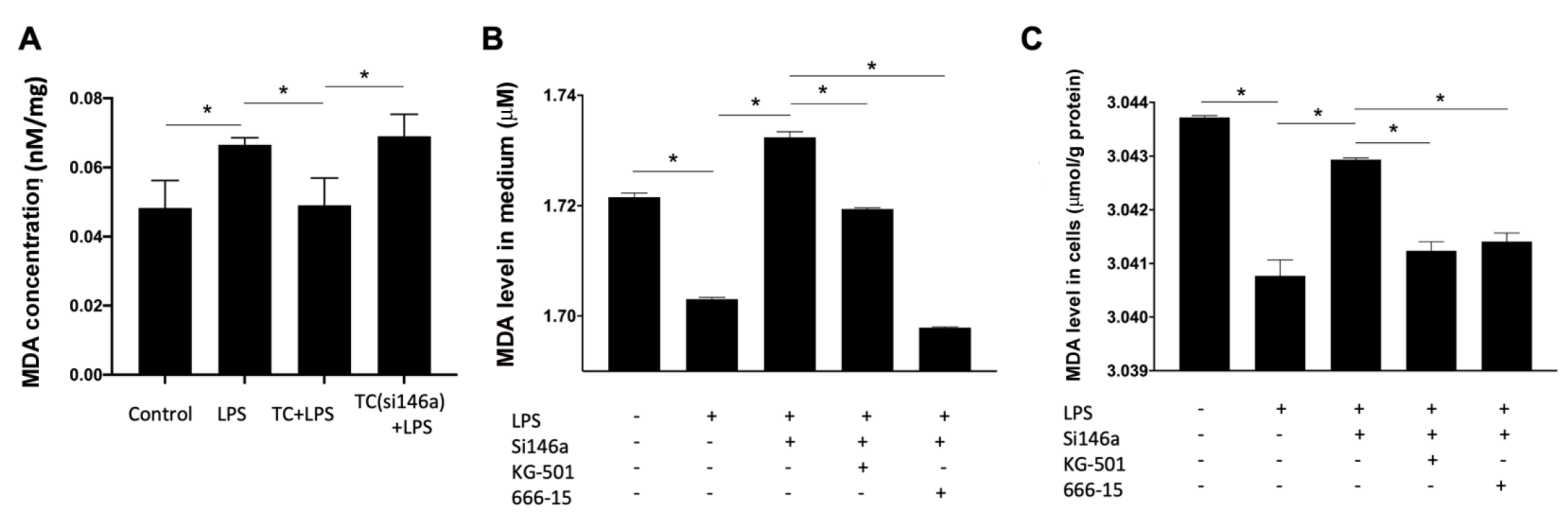
Figure2 MiR-146a-5p in TCs decreases oxidative stress both *in vitro* and *in vivo* (A) MDA levels in the lung tissue of ARDS mice were increased, and decreased in the telocyte-treated group. MDA levels were increased again when infused with telocytes pretreated with antagomiR-146a-5p. (B,C) MDA levels in the TCs and culture media were decreased when treated with LPS, and increased again when treated with antagomiR-146a-5p. Both KG-501 and 666-15 reduced the MDA level in TCs silenced with antagomiR-146a-5p. Data are shown as the mean±standard deviation from six independent experiments (n=6). *P< 0.05.

MDA is the product of lipid oxidative stress, and it is correlated with the severity of oxidative stress. The MDA level was found to be increased in the lung tissue of ARDS mice, but decreased in the TCs-treated group mice, and increased again when infused with TCs pretreated with antagomiR-146a-5p ( Figure 2A). MDA levels in TCs and culture media were decreased after treatment with LPS, and restored when being pretreated with antagomiR-146a-5p ( Figure 2B,C). Both KG-501 and 666-15 reduced the MDA level in TCs pretreated with antagomiR-146a-5p ( Figure 2B,C).

### MiR-146a-5p decreases oxidative stress through DUOX2 in TCs

**Figure 3:**
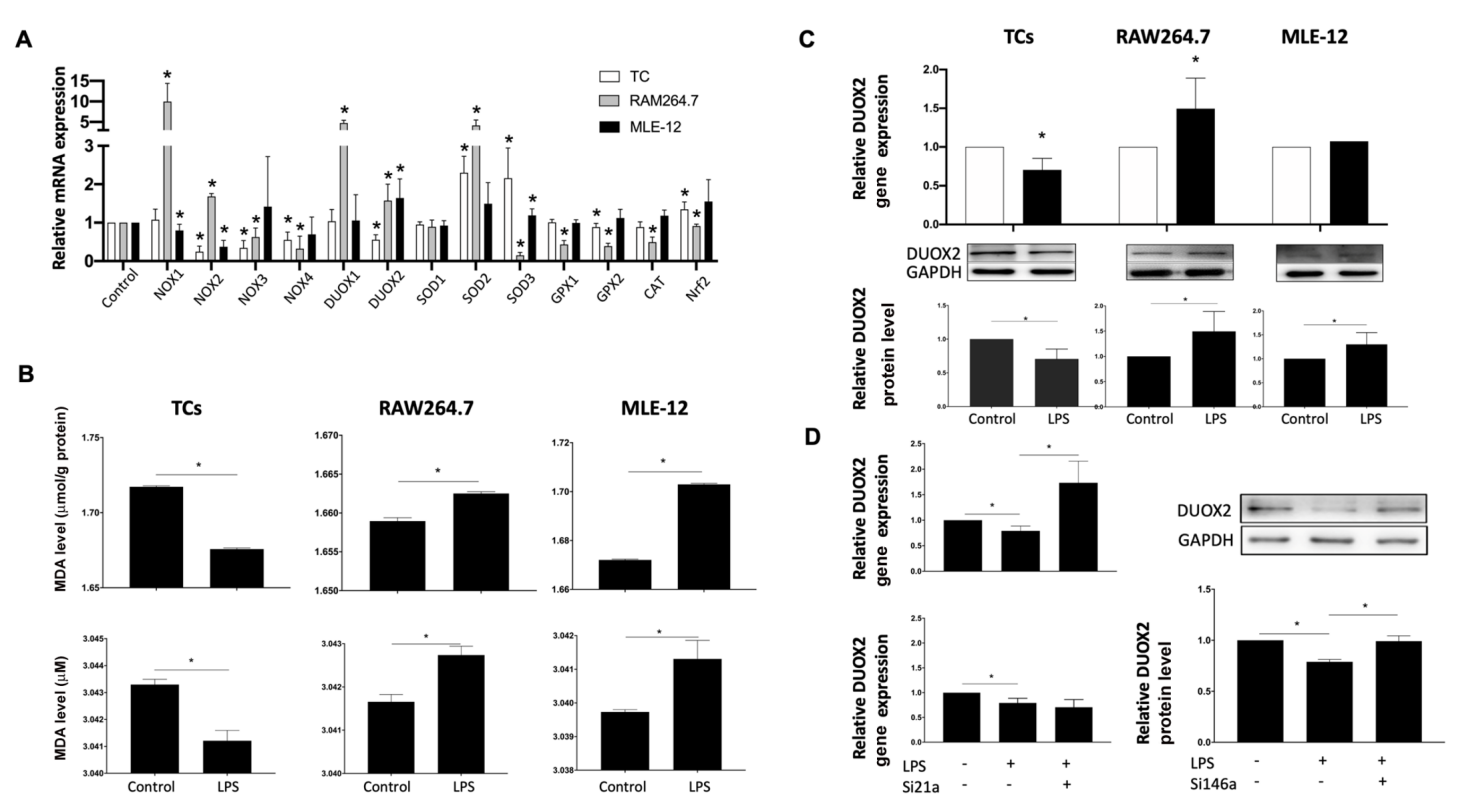
Figure3 MiR-146a-5p in telocytes decreases oxidative stress through the DUOX2 pathway (A) The screening results of the enzymes involved in TC protection. (B) LPS increased MDA levels in both RAW264.7 and MLE-12 cells but reduced MDA levels in TCs. (C) DUOX2 was downregulated in TCs after LPS stimulation at both the mRNA and protein levels compared with those in RAW264.7 and MLE-12 cells. (D) Silencing of miR-146a-5p, but not miR-21a-3p, upregulated DUOX2 mRNA and protein expressions in TCs stimulated with LPS. Data are shown as the mean±standard deviation from six independent experiments (n=6). *P< 0.05.

To find out the enzymes involved in TCs protection, both RAW264.7 and MLE-12 cells were used in the present study. RAW264.7 cells were accumulated in the lung of ARDS mice, while MLE-12 cells which are the pulmonary epithelial cells, were destroyed in ARDS mice. In the cultured RAW264.7 cells, LPS stimulation significantly increased mRNA expressions of oxidative enzyme NADPH oxidases 1 (NOX1), NOX2, dual oxidase 1 (DUOX1), and DUOX2, but significantly decreased mRNA expressions of antioxidant enzymes superoxide dismutase 3 (SOD3), glutathione peroxidase 1 (GPX1), GPX2, and catalase (CAT) ( Figure 3A). In MLE-12 cells, LPS significantly increased mRNA expressions of DUOX2 and SOD3, but significantly reduced the expressions of NOX1 and NOX2 ( Figure 3A). In TCs, LPS significantly increased SOD2 and SOD3 mRNA expressions, but significantly reduced NOX2, NOX3, NOX4, and DUOX2 mRNA expressions ( Figure 3A). Meanwhile, MDA levels were increased in both RAW264.7 and MLE-12 cells but reduced in TCs after stimulation with LPS ( Figure 3B). Since *DUOX2* is the candidate gene distinguishing TCs from RAW264.7 and MLE-12, DUOX2 protein expression was measured in the three cells stimulated with LPS. DUOX2 protein level was significantly increased in RAW264.7 and MLE-12 cells but significantly reduced in TCs after treatment with LPS ( Figure 3C). Silencing of miR-146a-5p, but not miR-21a-3p upregulated the mRNA and protein expressions of DUOX2 in TCs stimulated with LPS ( Figure 3D).

### MiR-146a-5p regulates the expression of DUOX2 through CREB1 in TCs

**Figure 4:**
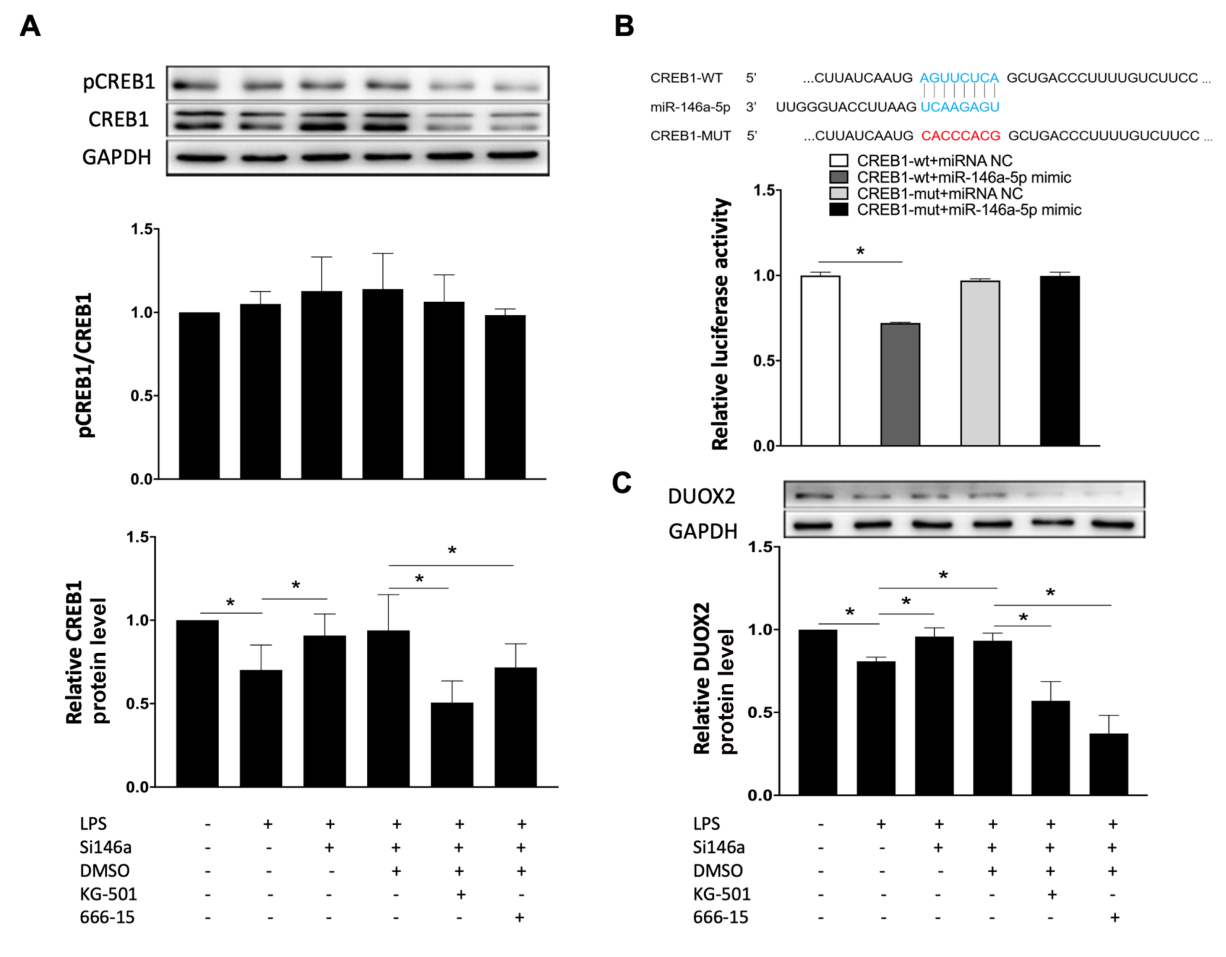
Figure4 Mir-146a-5p regulates the expression of DUOX2 through the CREB1 pathway (A) The expression of CREB1 in telocytes was decreased when treated with LPS and increased when TCs were treated with antagomiR-146a-5p. AntagomiR-146a-5p restored CREB expression in the presence of LPS. (B) A dual-luciferase reporter assay revealed that the miR-146a-5p mimic significantly reduced the CREB1 promoter activity. (C) AntagomiR-146a-5p significantly increased DUOX2 protein expression in TCs compared with cells transfected with the negative control. In the presence of antagomiR-146a-5p and LPS, KG-501 and 666-15 significantly reduced DUOX2 protein expression. Data are shown as the mean±standard deviation from six independent experiments (n=6). *P< 0.05.

The expression of CREB1 in TCs was decreased after treatment with LPS, and increased when TCs were pretreated with antagomiR-146a-5p. LPS significantly reduced CREB1 protein expression, both phosphorylated protein and total protein. AntagomiR-146a-5p restored CREB1 expression in the presence of LPS ( Figure 4A). Dual-luciferase reporter assay revealed that miR-146a-5p mimic significantly reduced wild-type *CREB1* promoter activity in HEK293T cells, but not in HEK293T cells with mutated *CREB1* ( Figure 4B). AntagomiR-146a-5p significantly increased DUOX2 protein expression in TCs when compared with cells transfected with the negative control. In the presence of antagomiR-146a-5p and LPS, the pharmacological inhibitors of CREB1, KG-501 and 666-15, significantly reduced DUOX2 protein expression ( Figure 4C).

### Family members of JAK/STAT are not involved in the expression of DUOX2 in TCs

**Figure 5:**
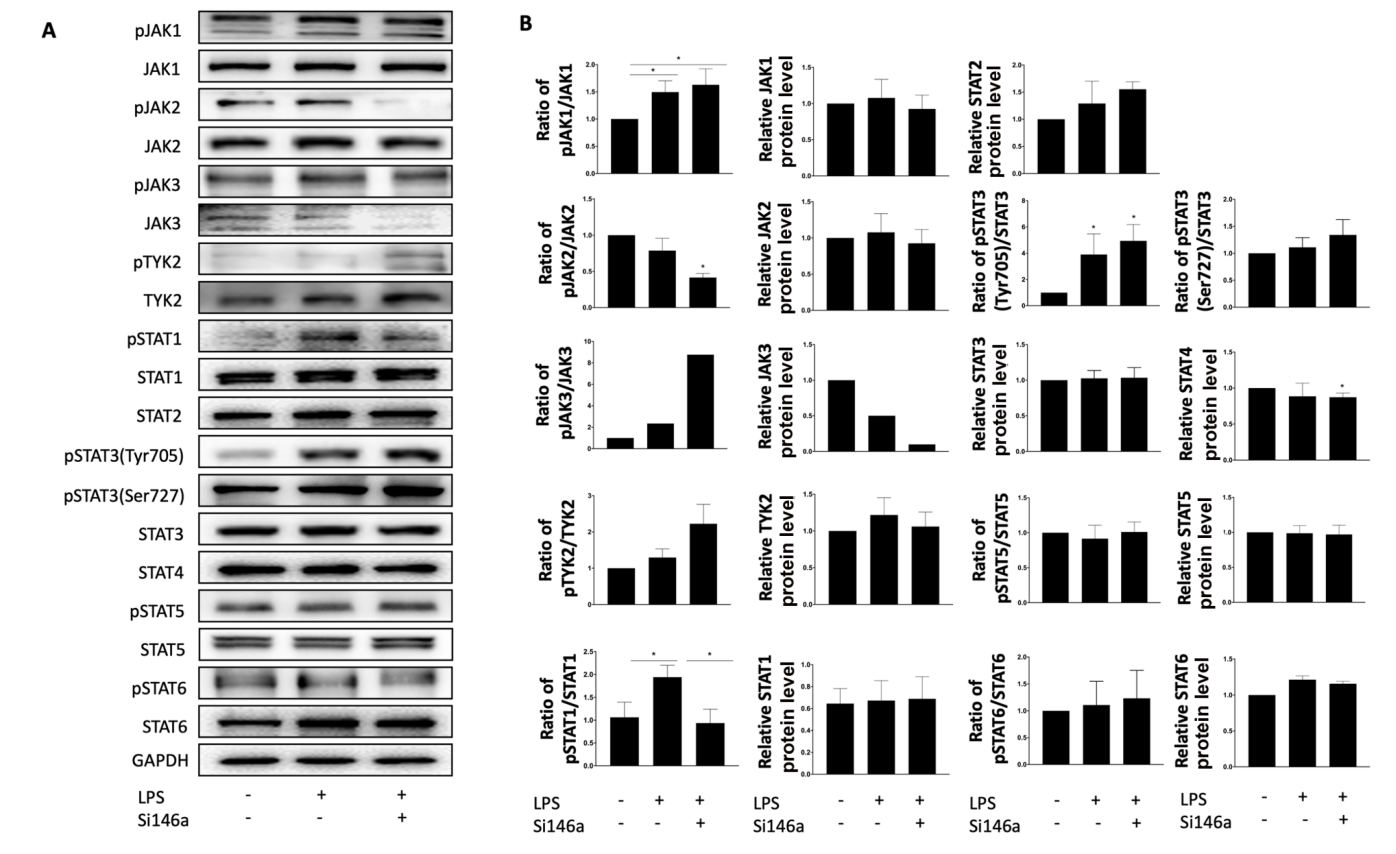
Figure5 Phosphorylation of family members of JAK/STAT are upregulated after LPS stimulation in telocytes but are not related to the expression of DUOX2 (A) LPS significantly increased the levels of phosphorylated JAK1, STAT1, and STAT3. (B) The levels of phosphorylated JAK1 and STAT3 failed to be restored by antagomiR-146a-5p incubation, but the level of phosphorylated STAT1 was restored. Data are shown as the mean±standard deviation from six independent experiments (n=6). *P< 0.05.

After stimulation with LPS for 6 h, the levels of phosphorylated JAK1 and STAT3(Tyr705) were significantly increased ( Figure 5A), but did not change after treatment with antagomiR-146a-5p ( Figure 5B). LPS stimulation increased level of phosphorylated STAT1 on tyrosine 701 residue ( Figure 5A), which was restored by antagomiR-146a-5p treatment, but it failed to change the expression of DUOX2 ( Figure 5B).

## Discussion

The present study reports that DUOX2 protein plays a vital role in the protective effect of TCs in the mouse ARDS model. After LPS stimulation, DUOX2 protein expression is decreased in mouse TCs, which is regulated by transcription factor CREB1. Enhanced expression of miR-146a-5p inhibits CREB1 transcription, resulting in reduced expression of DUOX2 protein as well as a decrease in oxidative stress.

The enzyme DUOX2, a member of the nicotinamide adenine dinucleotide phosphate (NADPH) family, has a peroxidase homology domain and a gp91phox domain [40]. Increased expression of DUOX2 has been reported in human pancreatic cancer cells when being exposed to IFN-γ [41]. In the present study, DUOX2 expression was found to be reduced in pulmonary TCs, but increased in RAW264.7 and MLE-12, suggesting that TCs exert distinguished antioxidative effects in response to LPS as well as in the mouse ARDS model.

DUOX2 protein is regulated at a transcriptional level. In cultured human pancreatic cells, interferon-gamma activates STAT1, with increased phosphorylation on tyrosine 701 residue, leading to the upregulation of DUOX2 protein [ 41, 42]. In the present study, we found that LPS stimulation increased level of phosphorylated STAT1 in TCs, but reduced DUOX2 expression, indicating that STAT1 does not regulate DUOX2 protein in TCs. CREB1 binds to the promoter of the DUOX2 gene. LPS stimulation reduced CREB1 protein expression, both the phosphorylated protein and total protein. Inhibition of expression of miR-146a-5p, in the presence of antagomiR-146a-5p in which CREB1 and DUOX2 expressions were restored, upregulated DUOX2 protein supporting the note that CREB1 regulates DUOX2 protein expression.

MicroRNAs (miRNAs), small non-coding ribonucleic acid molecules, regulate target genes by inhibiting mRNA translation or promoting mRNA degradation [ 43, 44]. It has been proposed that miRNAs are potential therapeutic targets in acute lung injury [45], since miR-216a level is decreased in ARDS patients, and the reduced expression of miR-216a is negatively correlated with the mortality of ARDS patients, while overexpressing miR-216a decreases TNF α, IL-6, and IL-1 β levels in adenocarcinomic human alveolar basal epithelial A549 cells stimulated with LPS [46]. Inhibition of miR-26a disrupts human lung microvascular endothelial cells barrier via increasing the expression of Eph receptors EA2 [47]. Neutrophil-derived miR-223 accelerates acute lung injury by downregulating poly(ADP-ribose)polymerase-1 (PARP-1) protein [48]. MicroRNA-146a alleviates LPS-mediated inflammation in a mouse ARDS model by inhibiting protein expressions of interleukin-1 receptor-associated kinase 1 and TNF receptor-associated factor 6 [ 49, 50]. MicroRNA-21a-3p promotes angiogenesis in endothelial cells under inflammatory conditions by regulating E2F8-Notch2 protein expressions [ 20, 28]. In the present study, miR-146a-5p is found to regulate CREB1 protein expression by binding with its promoter, leading to decreased expression of DUOX2 protein, indicating that miR-146a-5p exerts beneficial effects by downregulating oxidative enzymes in TCs.

**Figure 6:**
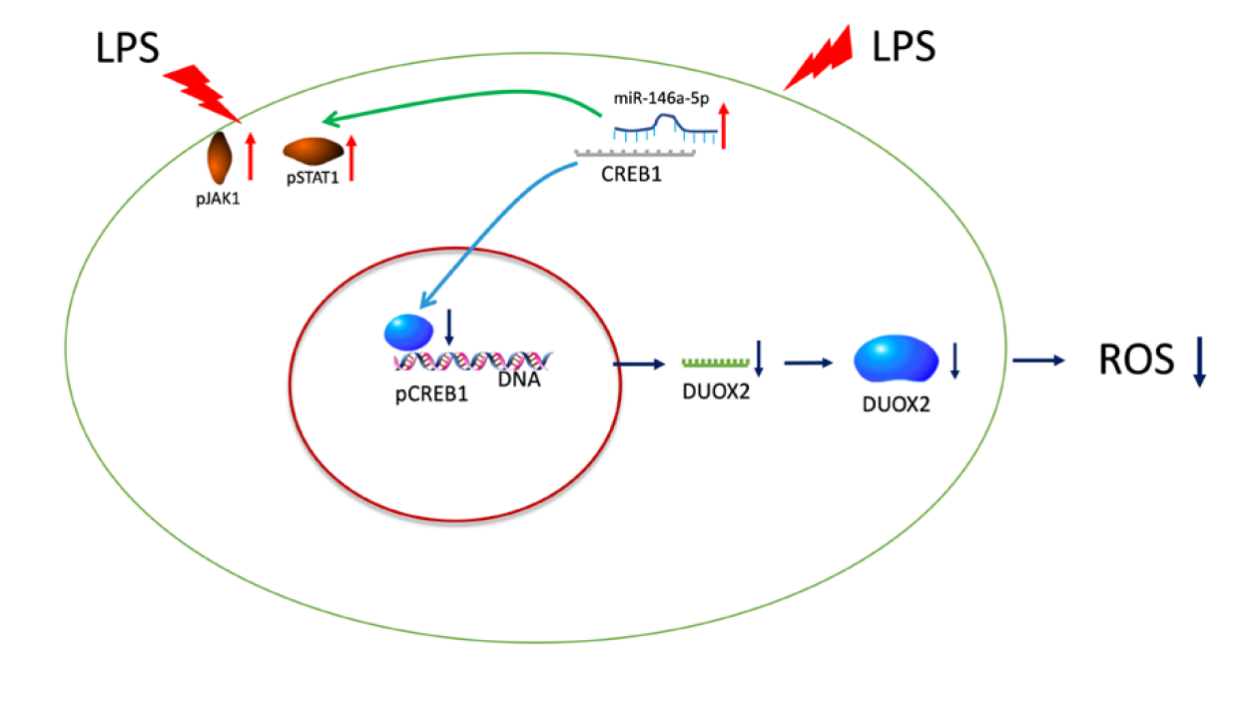
Figure6 Schematic diagram of the pathway of DUOX2 production controlled by miR-146a-5p LPS-induced DUOX2 production was decreased through the miR-146a-5p/CREB1/DUOX2 signaling pathway in TCs. TC, telocyte; ROS, reactive oxygen species; LPS, lipopolysaccharide; JAK, janus kinase; STAT, signal transducer and activator of transcription; DUOX2, dual oxidase 2; and CREB1, cAMP-response element binding protein 1.

In conclusion, miR-146a-5p in TCs downregulates the expression of DUOX2 via the CREB1 pathway and administration of TCs protects the function of the lung undergoing oxidative stress damage ( Figure 6).
